# Complicated appendicitis and surgical site infection associated with prolonged hospital stay after appendectomy in a Peruvian hospital

**DOI:** 10.1016/j.sopen.2026.08.004

**Published:** 2026-09-02

**Authors:** Irene A. Arias-Romaní, Miguel A. Arce-Huamani

**Affiliations:** a Programa Academico de Medicina Humana, Facultad de Ciencias de la Salud, Universidad Privada Norbert Wiener, Lima, 15046, Peru

**Keywords:** Acute appendicitis, Appendectomy, Length of stay, Complicated appendicitis, Surgical site infection

## Abstract

**Objective:**

To identify factors associated with prolonged hospital stay after appendectomy for acute appendicitis in a Peruvian public hospital.

**Methods:**

We conducted a retrospective case-control study of adults who underwent appendectomy for acute appendicitis at Hospital Jesús Nazareno, Ayacucho, Peru, between January 2021 and December 2024. Cases had a hospital stay of ≥5 days, corresponding to the 75th percentile of the institutional distribution, and controls had a stay of <5 days. From 96 eligible cases and 288 eligible controls, 73 patients from each group were selected by simple random sampling. Crude and adjusted odds ratios (ORs) with 95% confidence intervals (CIs) were estimated using logistic regression.

**Results:**

Median hospital stay was 6.0 days (IQR, 5.0–9.0) among cases and 2.0 days (IQR, 2.0–3.0) among controls. Complicated appendicitis was more frequent among cases than controls (97.3% vs. 27.4%), as was surgical site infection (38.4% vs. 1.4%). After multivariable adjustment, complicated appendicitis (adjusted OR, 38.62; 95% CI, 7.78–191.82) and surgical site infection (adjusted OR, 9.58; 95% CI, 1.14–80.55) remained associated with prolonged hospital stay.

**Conclusion:**

Complicated appendicitis and surgical site infection were associated with prolonged hospital stay after appendectomy. Because the timing of surgical site infection onset was not captured, its temporal relationship with prolonged stay cannot be established. Timely management of complicated appendicitis and adherence to perioperative infection-prevention practices may help reduce prolonged hospitalization.

## Graphical abstract

**Figure:**
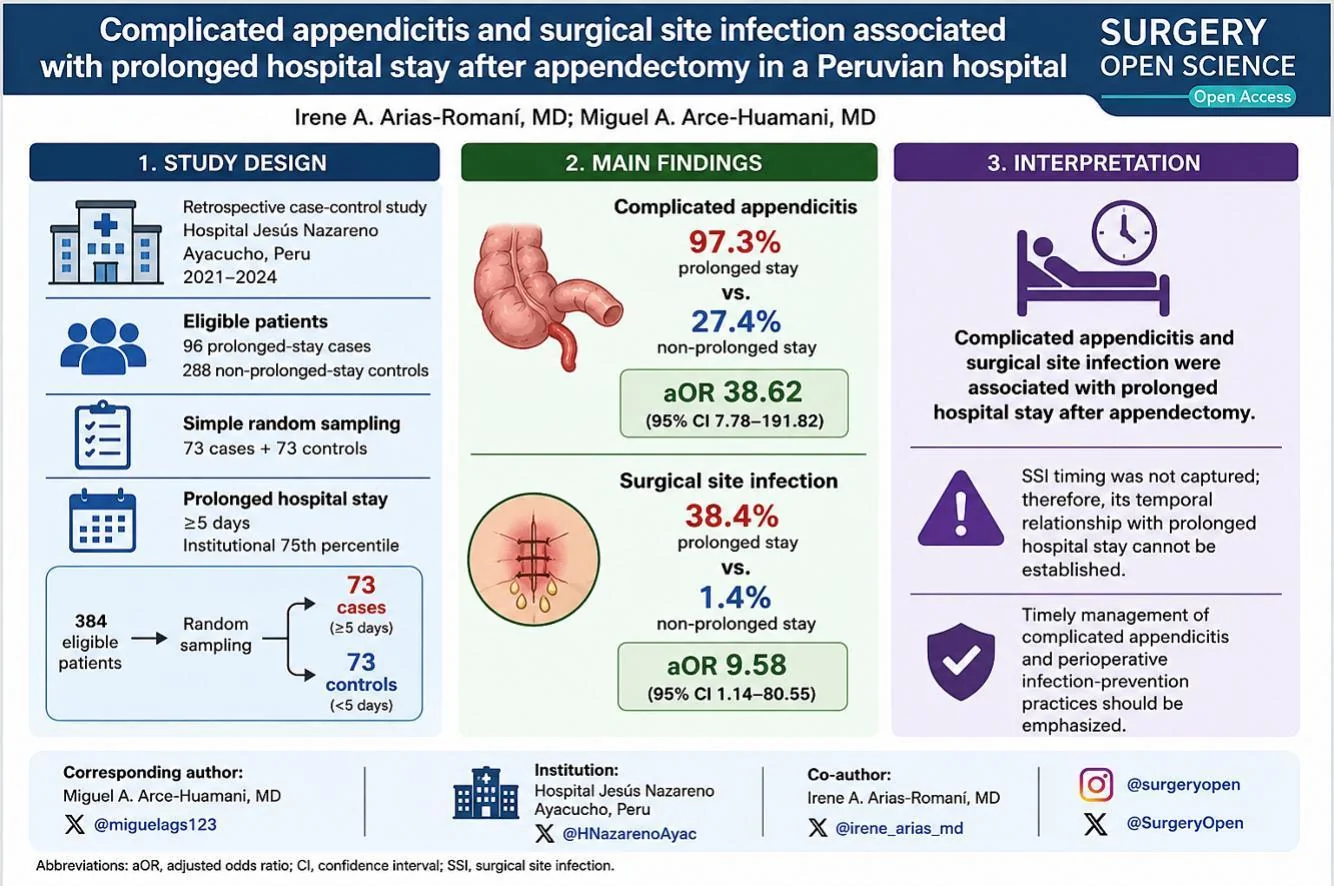

## Introduction

Acute appendicitis remains one of the most frequent emergency abdominal conditions worldwide, with an estimated lifetime incidence of approximately 7–8% in Western populations and a clinical spectrum that ranges from localized inflammation to perforation, abscess, peritonitis, and postoperative infectious complications [1], [2]. Appendectomy continues to be a definitive treatment for acute appendicitis and is generally considered a safe procedure, although reported mortality still ranges from 0.09% to 0.24% depending on patient and disease characteristics [3], [4]. Beyond mortality, postoperative length of stay is increasingly used as an outcome that captures clinical severity, recovery trajectory, resource consumption, and hospital performance after surgical care [5], [6]. Thus, prolonged hospitalization after appendectomy represents both a surgical and health-system problem.

Disease severity is one of the main factors associated with delayed discharge after appendectomy [1], [7]. In a cohort of 449 adults with acute appendicitis, Alotaibi et al. reported that complicated appendicitis represented nearly one fifth of cases and was independently associated with longer hospitalization, with a hazard ratio of 6.61 for increased hospital stay [8]. In a larger cohort of 1638 adults undergoing emergency appendectomy, Bancke Laverde et al. found that prolonged stay was associated with older age, elevated inflammatory markers, lower hemoglobin levels, conversion to open surgery, longer operative time, complicated appendicitis, and postoperative morbidity [9].

Evidence from patients with uncomplicated appendicitis suggests a somewhat different pattern. Among 497 patients treated by laparoscopic appendectomy, Shin et al. reported that late discharge, defined as a postoperative length of stay exceeding 2 days, occurred in 7.4% of cases. Although age, preoperative C-reactive protein levels, and operative time differed between the early- and late-discharge groups, only operative time remained associated with prolonged hospitalization after multivariable adjustment [10]. These findings suggest that the factors associated with prolonged stay may differ between uncomplicated and complicated appendicitis.

Postoperative management and infectious complications are also central to hospital stay after appendectomy. In patients with complex appendicitis receiving postoperative antibiotics, van den Boom et al. reported that discharge criteria were reached after a median of 2 days, while antibiotic duration and hospital stay both had a median of 5 days, and infectious complications occurred in 12% of patients [11]. In adults with complicated acute appendicitis, Martínez-Pérez et al. identified postoperative complications and abdominal drainage as factors associated with prolonged length of stay after laparoscopic appendectomy [12]. More recently, Alyhari et al. evaluated 245 consecutive open appendectomy cases and developed a risk prediction model for surgical site infection using routinely available clinical variables [13]. These findings reinforce that discharge after appendectomy depends not only on removal of the appendix, but also on infection control, postoperative surveillance, and clinical stability.

Evidence from Peru remains limited, although available data indicate that postoperative outcomes after appendectomy are clinically relevant in the national context. A Peruvian systematic review including 1766 patients who underwent appendectomy reported that 53.45% were male, surgical site infection occurred in 7%, and the mean hospital stay was 3.67 days [14]. In Lima, a matched case-control study found that complicated appendicitis was associated with SARS-CoV-2 infection, with an adjusted conditional OR of 3.52 [15].

Enhanced recovery strategies have been proposed to reduce postoperative stay and costs after laparoscopic appendectomy, but their implementation depends on disease severity, institutional resources, and postoperative safety [6]. Local evidence from Andean hospitals is therefore needed to identify the clinical and surgical factors associated with prolonged hospitalization after appendectomy.

This study aimed to identify factors associated with prolonged hospital stay after appendectomy for acute appendicitis in a Peruvian hospital between 2021 and 2024.

## Materials and methods

### Study design and setting

We conducted a quantitative, observational, analytical, retrospective case-control study among patients who underwent surgery for acute appendicitis in Peru. The study was carried out at Hospital Jesús Nazareno, a public hospital located in Ayacucho, Peru. Clinical records of patients treated between January 2021 and December 2024 were reviewed. The study was designed to identify clinical, surgical, and postoperative factors associated with prolonged hospital stay after appendectomy for acute appendicitis.

### Study population, sample size, and sampling

The study population consisted of adult patients who underwent appendectomy for acute appendicitis at Hospital Jesús Nazareno of Ayacucho during the study period. Patients were classified according to hospital stay. The 75th percentile of the institutional hospital-stay distribution corresponded to 5 days. Accordingly, cases were patients with a hospital stay of ≥5 days, whereas controls were patients with a hospital stay of <5 days.

The sample size was calculated for an analytical case-control design, assuming a 95% confidence level, 80% statistical power, and a 1:1 case-to-control ratio. The calculation assumed an expected exposure prevalence of 20% among controls and an odds ratio of 2.8, based on previous evidence on factors associated with prolonged hospital stay after appendectomy [16]. The minimum required sample size was 146 patients, comprising 73 cases and 73 controls.

The eligible sampling frame comprised 96 patients with prolonged hospital stay and 288 patients with non-prolonged hospital stay. From these eligible groups, 73 cases and 73 controls were selected by simple random sampling to achieve the prespecified 1:1 ratio. The final analytical sample therefore included 146 patients with complete information for the outcome and the covariates included in the primary multivariable model. Missing C-reactive protein measurements did not result in exclusion because CRP was evaluated only descriptively using available measurements. Because sampling was conditioned on hospital-stay status, the combined analytical sample was not intended to reproduce the distribution of prolonged and non-prolonged hospital stay in the underlying hospital population.

### Eligibility criteria

Patients were eligible for inclusion as cases if they were 18 years of age or older, had a confirmed diagnosis of acute appendicitis, underwent appendectomy at Hospital Jesús Nazareno of Ayacucho during 2021–2024, had prolonged hospital stay according to the operational definition, and had complete information for the outcome and the covariates included in the primary multivariable model.

Patients were eligible for inclusion as controls if they were 18 years of age or older, had a confirmed diagnosis of acute appendicitis, underwent appendectomy at the same hospital during the same period, had non-prolonged hospital stay according to the operational definition, and had complete information for the outcome and the covariates included in the primary multivariable model.

Patients were excluded if they underwent additional surgical procedures simultaneously with appendectomy, were referred from another health facility after previous surgical management, had voluntary discharge or transfer to another institution before completing treatment, or died during hospitalization from causes unrelated to appendectomy. Clinical records with missing information for the outcome or any covariate included in the primary multivariable model were excluded. Missing C-reactive protein measurements did not result in exclusion because CRP was not included in the primary adjusted model.

### Variables and definitions

The outcome variable was prolonged hospital stay among patients undergoing appendectomy for acute appendicitis. Hospital stay was defined as the number of calendar days from hospital admission to discharge during the index hospitalization in which appendectomy was performed. The 75th percentile of the institutional hospital-stay distribution corresponded to 5 days. Accordingly, prolonged hospital stay was defined as a stay of ≥5 days, whereas a stay of <5 days was classified as non-prolonged.

The explanatory variables were grouped into demographic, anthropometric, preoperative clinical, surgical, and postoperative factors. Demographic and anthropometric variables included age, sex, body mass index, and obesity. Preoperative clinical variables included type 2 diabetes mellitus, hypertension, harmful habits, hemoglobin level, leukocyte count, and C-reactive protein (CRP). Laboratory variables were reported using SI units: hemoglobin in g/L, leukocyte count in ×10^9^/L, and CRP in mg/L. Surgical and postoperative variables included type of appendicitis, operative time, surgical site infection (SSI), pneumonia, and sepsis.

Type of appendicitis was categorized as non-complicated or complicated according to the diagnosis documented in the clinical record. Complicated appendicitis included gangrenous or perforated appendicitis, periappendiceal abscess, and localized or generalized peritonitis. Non-complicated appendicitis comprised inflammatory forms without gangrene, perforation, abscess, or peritonitis. Operative time was recorded in minutes and corresponded to the interval from the beginning of the surgical procedure to completion of wound closure, as documented in the operative record.

Surgical site infection was defined as a postoperative infection involving the operated anatomical site and documented in the clinical record on the basis of local findings such as erythema, warmth, pain, or purulent drainage. SSI was extracted as a dichotomous variable (yes/no). The data-collection form did not capture the date of SSI onset or diagnosis; therefore, its temporal relationship to surgery, the 5-day threshold for prolonged hospital stay, and hospital discharge could not be determined from the study dataset. Post-discharge SSI surveillance was not captured in the study dataset. Pneumonia and sepsis were likewise recorded as dichotomous postoperative complications according to their documentation in the clinical record.

CRP was available as a numerical measurement for only 26 of the 146 patients (17.8%) and was therefore summarized descriptively on a complete-case basis. No missing values were imputed. Because 120 of 146 CRP observations (82.2%) were missing and availability was highly selective, no inferential between-group test was performed for CRP, and this variable was not included in the multivariable model.

### Data collection procedures

Data were collected through documentary review of clinical records using a structured data extraction form prepared for the study. The form included all variables defined in the operational matrix and was designed to standardize the extraction of demographic, clinical, laboratory, surgical, and postoperative information.

Before data collection, the instrument was reviewed by experts to assess relevance, clarity, and appropriateness for the study objectives. A pilot review of 20 clinical records was also performed to evaluate inter-rater reliability, obtaining a Cohen's kappa coefficient of 0.894, which indicated high agreement.

After authorization from the hospital, eligible clinical records were identified and selected according to the sampling procedure. The extracted information was entered into a coded database. The database was reviewed for completeness, consistency, and possible data entry errors before statistical analysis. No direct contact with patients was required because the study was based exclusively on retrospective review of clinical records.

### Statistical analysis

Categorical variables were summarized as frequencies and percentages. Continuous variables were summarized as means with standard deviations (SDs) when their distributions were approximately normal and as medians with interquartile ranges (IQRs) otherwise. Distributional assumptions were assessed separately within the prolonged- and non-prolonged-stay groups using the Shapiro–Wilk test together with visual inspection of histograms and normal Q–Q plots. The graphical assessment also considered skewness, multimodality, and potentially influential outliers. Homogeneity of variances was evaluated using Levene's test before parametric comparisons.

Between-group comparisons of categorical variables were performed using Pearson's chi-square test or Fisher's exac*t-*test, according to the expected cell frequencies. For continuous variables, the independent-samples Student's *t-*test was used when graphical exploration supported approximate normality and variances were homogeneous. Welch's t-test was planned for approximately normal variables with unequal variances, whereas the Mann–Whitney *U* test was used for non-normally distributed variables. Based on the combined graphical and formal assessment, BMI was compared using Student's *t-*test, while age, hemoglobin, leukocyte count, and operative time were compared using the Mann–Whitney *U* test. No inferential test was performed for CRP because numerical measurements were available for only 17.8% of the analytical sample and the missingness pattern was highly selective. Test selection was not based exclusively on the *p* value from the normality test [17]. All tests were two-sided, and a *p* value <0.05 was considered statistically significant.

**Supplementary Fig. 1:**
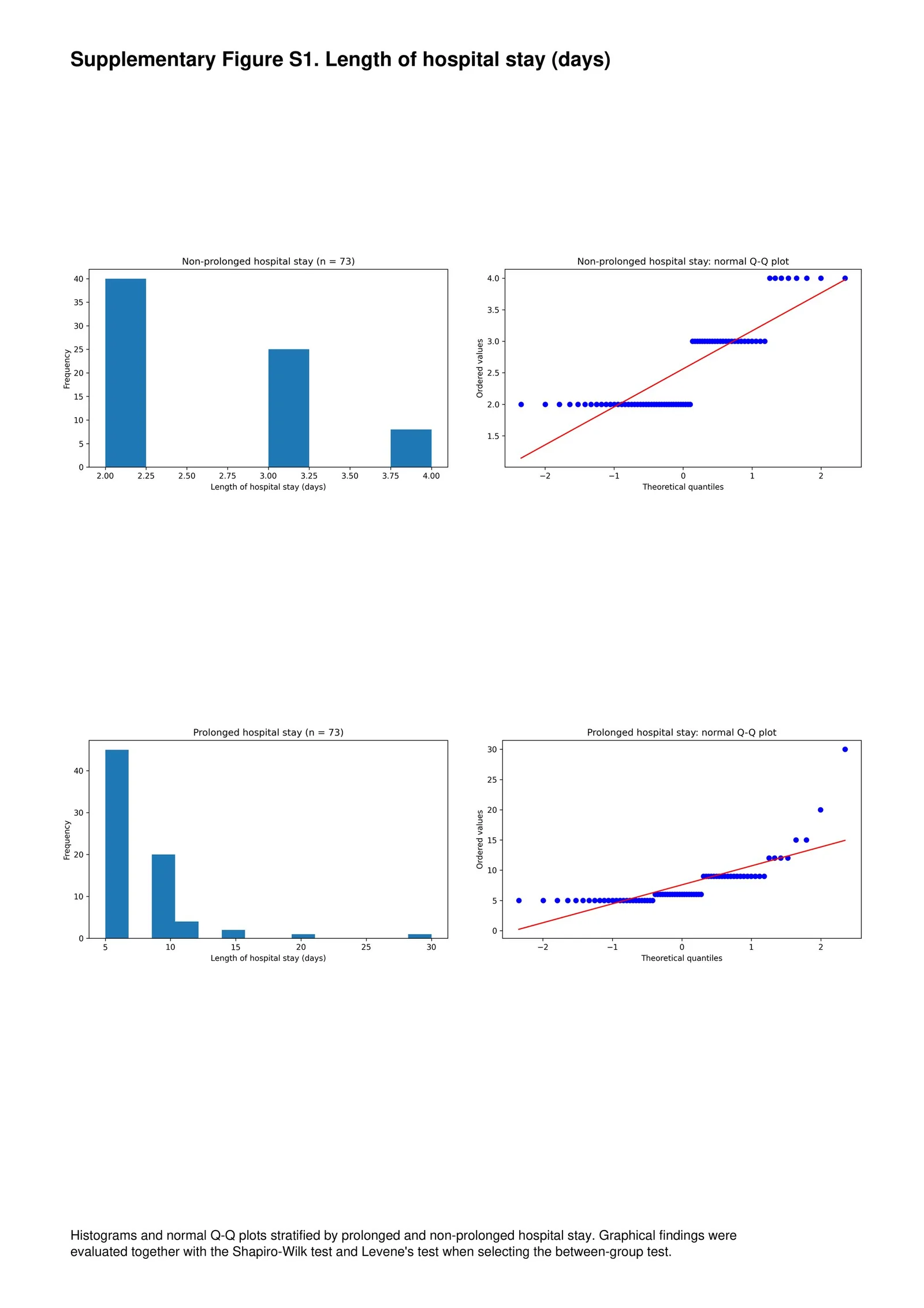
Length of hospital stay (days). Histograms and normal Q–Q plots stratified by prolonged and non-prolonged hospital stay. Graphical findings were evaluated together with the Shapiro–Wilk test and Levene’s test when selecting the between-group test.

**Supplementary Fig. 2:**
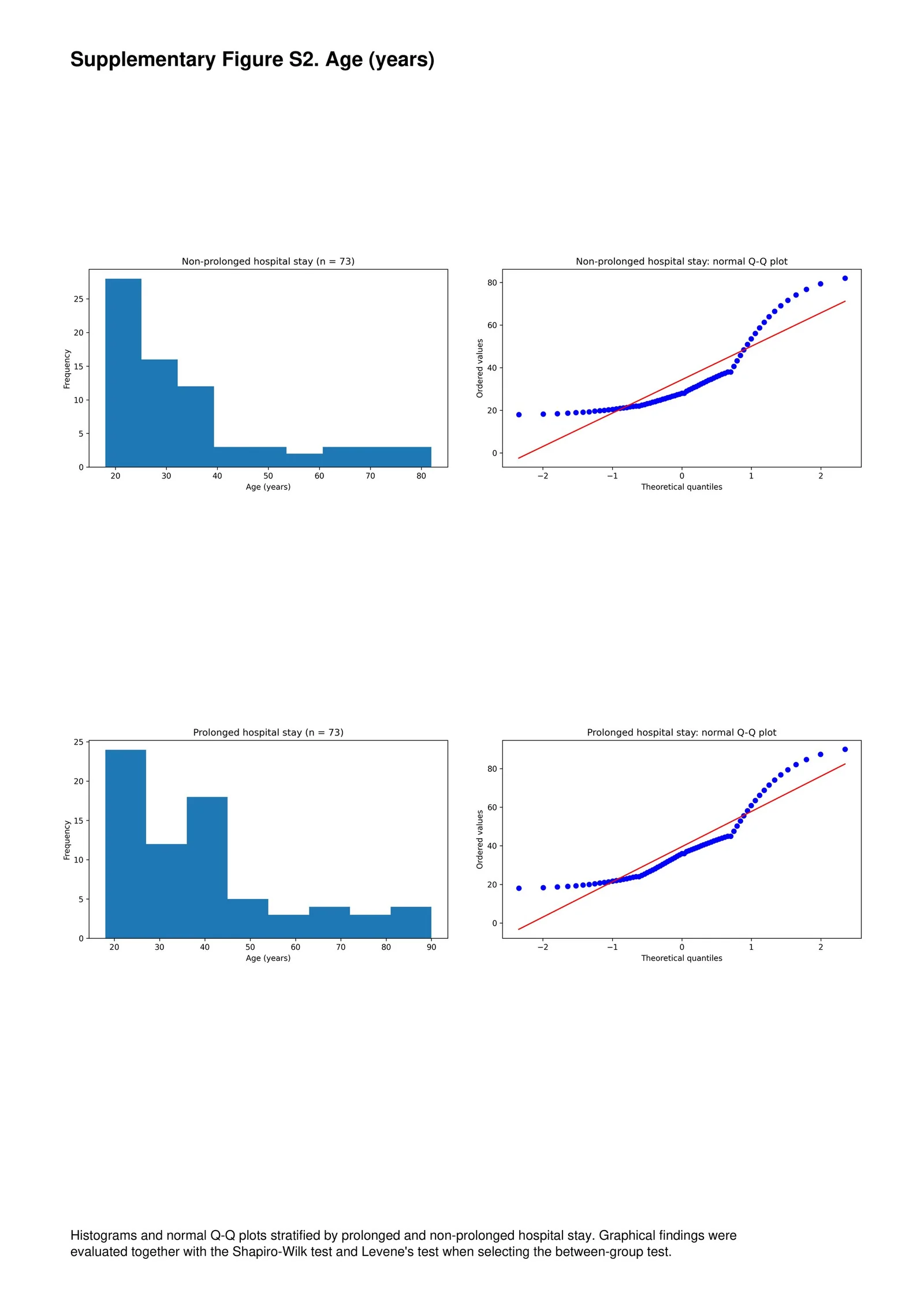
Age (years). Histograms and normal Q–Q plots stratified by prolonged and non-prolonged hospital stay. Graphical findings were evaluated together with the Shapiro–Wilk test and Levene’s test when selecting the between-group test.

**Supplementary Fig. 3:**
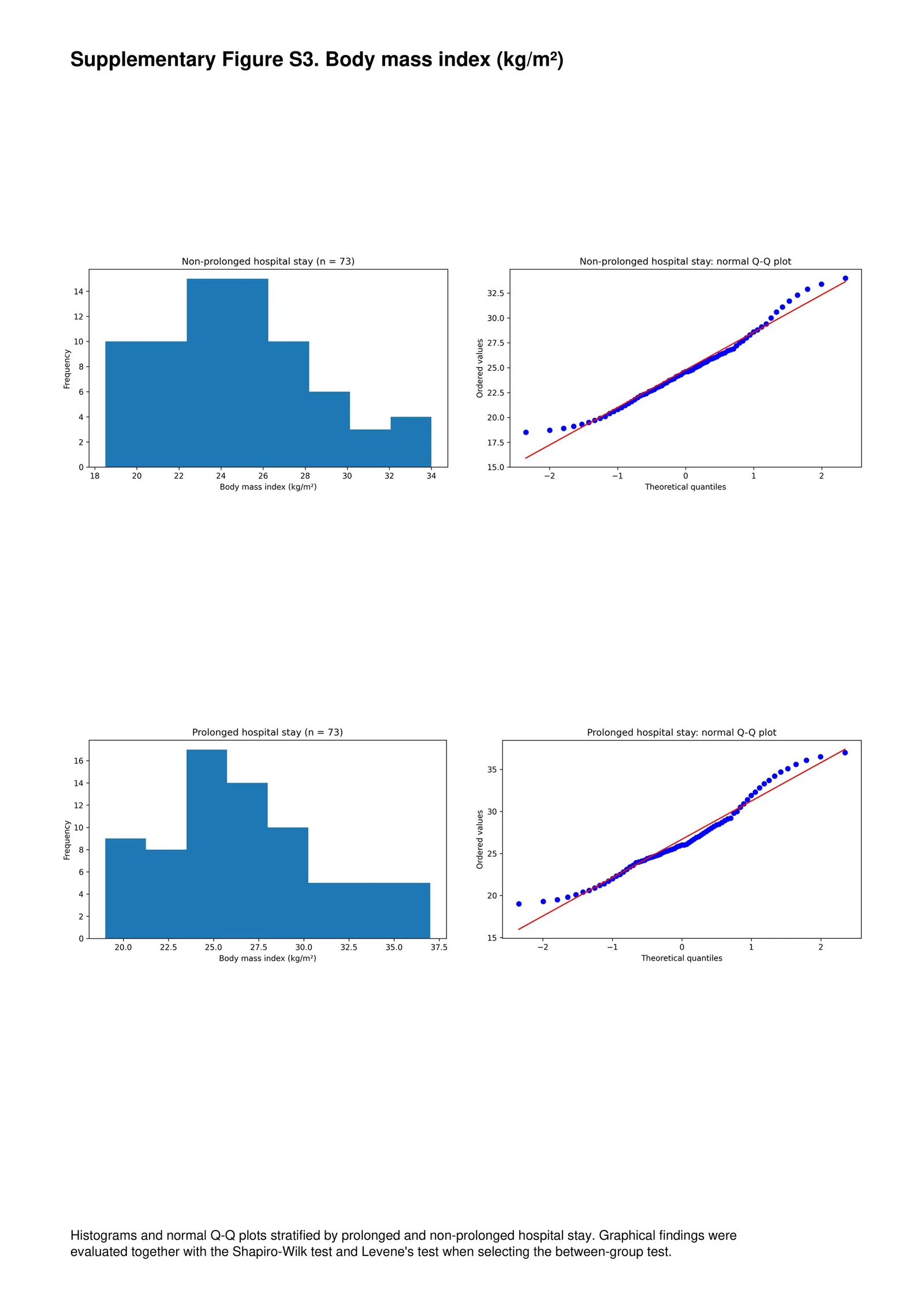
Body mass index (kg/m²). Histograms and normal Q–Q plots stratified by prolonged and non-prolonged hospital stay. Graphical findings were evaluated together with the Shapiro–Wilk test and Levene’s test when selecting the between-group test.

**Supplementary Fig. 4:**
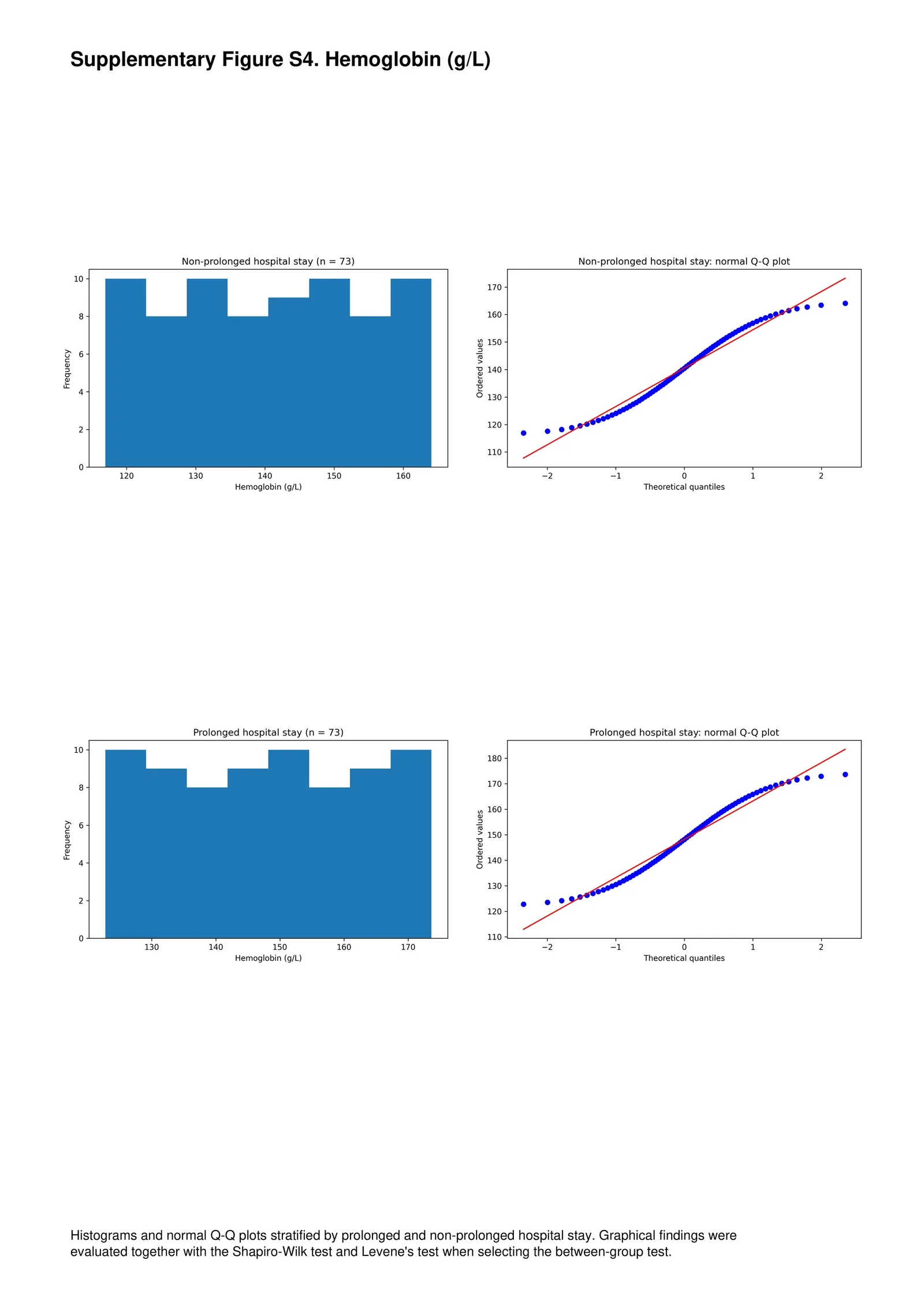
Hemoglobin (g/L). Histograms and normal Q–Q plots stratified by prolonged and non-prolonged hospital stay. Graphical findings were evaluated together with the Shapiro–Wilk test and Levene’s test when selecting the between-group test.

**Supplementary Fig. 5:**
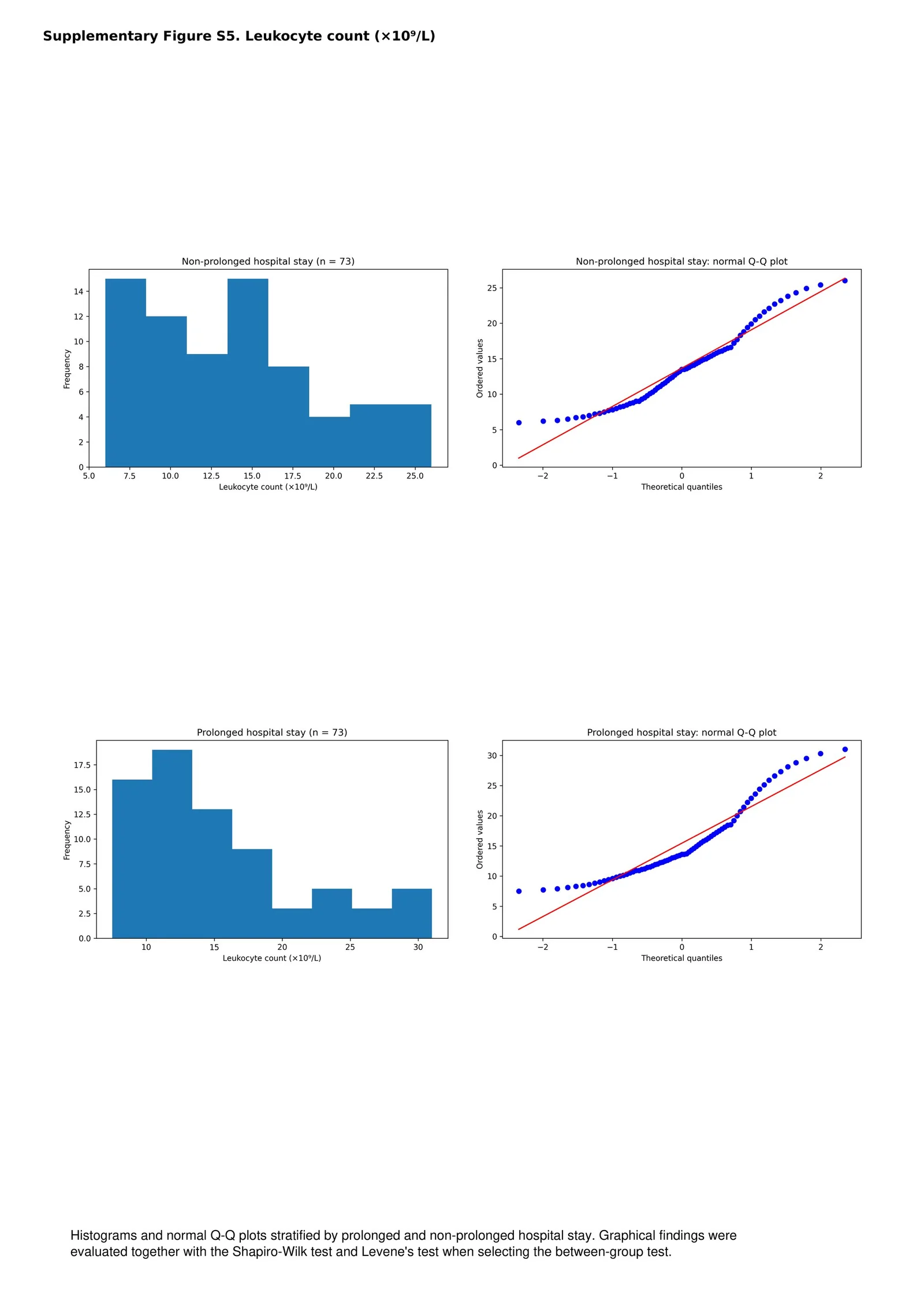
Leukocyte count (×10⁹/L). Histograms and normal Q–Q plots stratified by prolonged and non-prolonged hospital stay. Graphical findings were evaluated together with the Shapiro–Wilk test and Levene’s test when selecting the between-group test.

**Supplementary Fig. 6:**
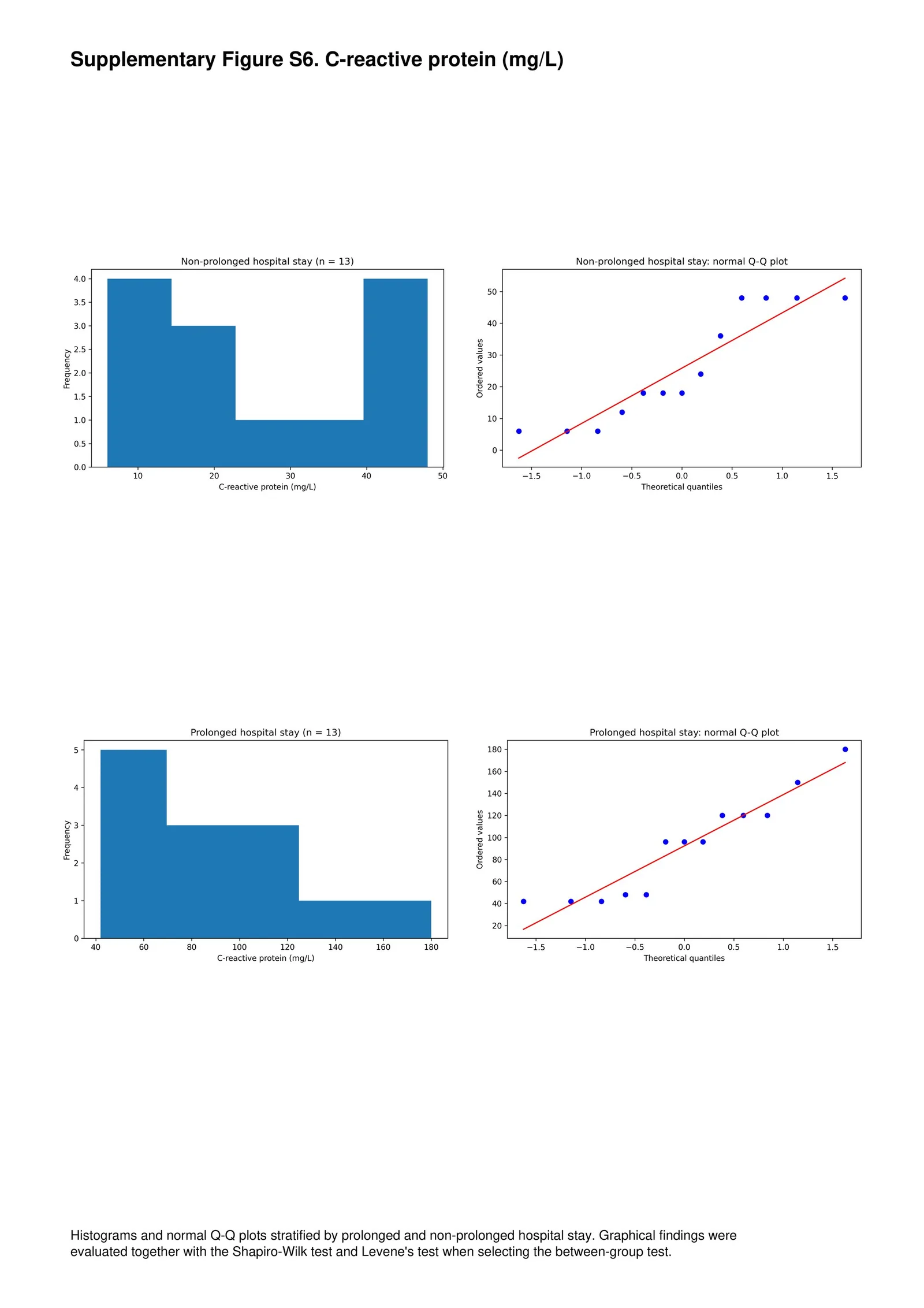
C-reactive protein (mg/L). Histograms and normal Q–Q plots stratified by prolonged and non-prolonged hospital stay. Graphical findings were evaluated together with the Shapiro–Wilk test and Levene’s test when selecting the between-group test.

**Supplementary Fig. 7:**
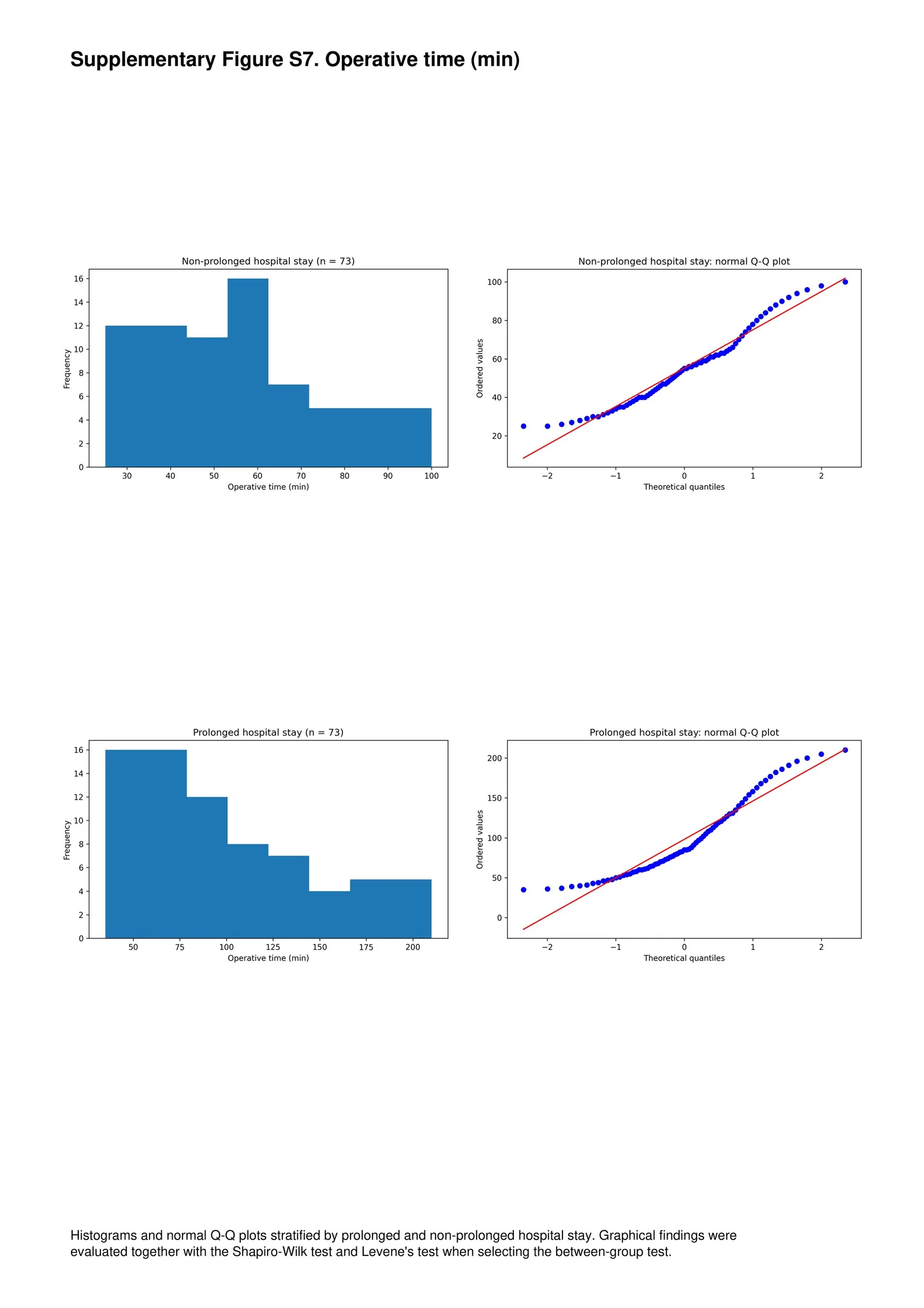
Operative time (min). Histograms and normal Q–Q plots stratified by prolonged and non-prolonged hospital stay. Graphical findings were evaluated together with the Shapiro–Wilk test and Levene’s test when selecting the between-group test.

The corresponding histograms and normal Q-Q plots are provided in Supplementary Figs. S1–S7, while the formal distributional assessment and the selected between-group tests are summarized in Supplementary Table S2.

The extent and pattern of missing C-reactive protein data were examined by reporting the number and percentage of available and missing measurements overall and within each hospital-stay group. CRP availability was examined according to hospital-stay group and other patient characteristics. Numerical CRP measurements were available for 26 patients (17.8%) and missing for 120 (82.2%). Because availability was highly selective, CR*P* values were summarized descriptively without an inferential between-group test. No imputation was performed.

Crude odds ratios with 95% confidence intervals were estimated using bivariate logistic regression models. The logistic regression analyses were intended to estimate associations rather than to develop a clinical prediction model. Accordingly, ORs were interpreted as measures of association. Model discrimination, calibration, clinical utility, and internal or external validation were not evaluated [18]. Variables with statistical significance in the bivariate analysis and variables considered clinically relevant were evaluated for inclusion in the multivariable logistic regression model. Adjusted odds ratios with 95% confidence intervals were calculated to estimate the associations between the selected covariates and prolonged hospital stay after multivariable adjustment.

Because the conventional adjusted ORs for complicated appendicitis and surgical site infection had wide confidence intervals, suggesting possible sparse-data instability, we performed an L2 ridge-penalized logistic regression sensitivity analysis using the same six covariates as the primary adjusted model: sex, age, hemoglobin level, type of appendicitis, surgical site infection, and operative time. Continuous covariates were standardized to a mean of 0 and a standard deviation of 1 before penalization, whereas binary indicators retained their 0/1 coding. The model was fitted with an unpenalized intercept and a regularization parameter of C = 1.0. Penalized ORs were back-transformed to their original reporting scales.

Uncertainty was evaluated using a stratified nonparametric bootstrap with 2000 resamples. Within each bootstrap replicate, 73 cases and 73 controls were sampled separately with replacement to preserve the case-control sampling ratio; continuous covariates were re-standardized within each bootstrap sample before model fitting. Percentile-based 95% bootstrap confidence intervals were then calculated from the resulting penalized estimates. A fixed random seed of 123 was used for reproducibility. To evaluate sensitivity to the degree of penalization, the model was additionally refitted using C values of 0.25, 0.50, 1.00, 2.00, and 4.00. This analysis was used to assess the stability of the direction and magnitude of the associations under penalization and was not interpreted as confirmation of the conventional adjusted estimates.

The primary adjusted model included sex, age, hemoglobin level, type of appendicitis, surgical site infection, and operative time. C-reactive protein was excluded because of substantial missingness, whereas pneumonia and sepsis were excluded because of their very low event counts. The outcome was coded as 1 for prolonged hospital stay and 0 for non-prolonged hospital stay; therefore, ORs greater than 1 indicated higher odds of prolonged stay.

**Fig. 1:**
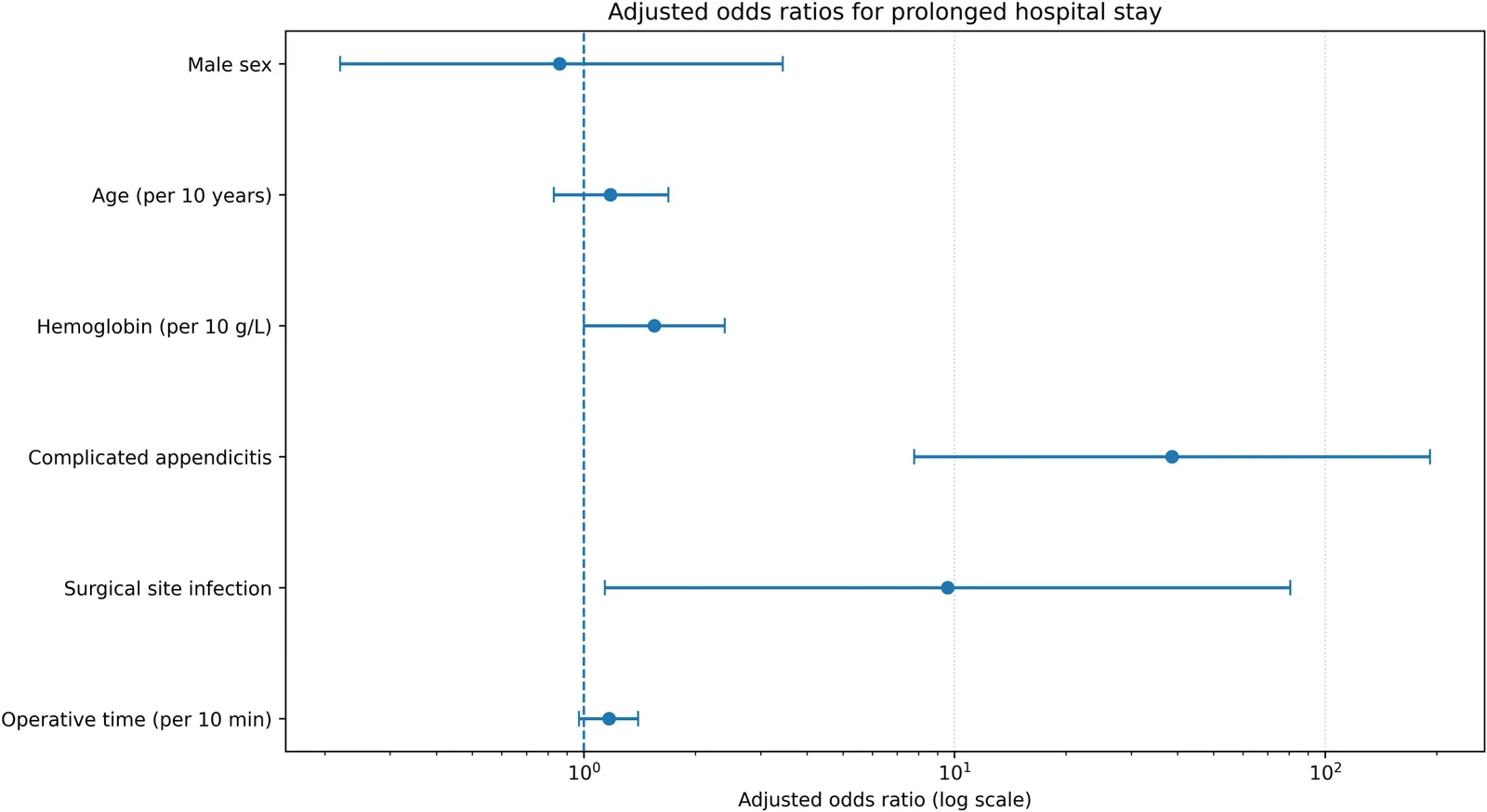
Adjusted odds ratios and 95% confidence intervals for prolonged hospital stay. The vertical dashed line represents the null value of OR = 1.

The descriptive, bivariate, and conventional logistic regression analyses were performed using IBM SPSS Statistics version 27 (IBM Corp., Armonk, NY, USA). The ridge-penalized sensitivity analysis was performed using Python version 3.13.5 and scikit-learn version 1.8.0, using LogisticRegression with an L2 penalty, an unpenalized intercept, C = 1.0 for the primary sensitivity analysis, and the lbfgs solver. Additional analyses were performed using C values of 0.25, 0.50, 2.00, and 4.00. Fig. 1 was generated using Python version 3.13.5 and Matplotlib version 3.10.8. All tests were two-sided, and *p* < 0.05 was considered statistically significant.

### Ethical considerations

The study was conducted in accordance with the ethical principles of the Declaration of Helsinki and applicable Peruvian regulations for health research. The protocol was reviewed and approved by the Institutional Committee of Ethics and Scientific Integrity of Universidad Privada Norbert Wiener, Lima, Peru, under approval file No. 0714–2026. The approved protocol was titled “Factores asociados a la estancia hospitalaria prolongada en postoperados por apendicitis aguda en un hospital de Ayacucho, 2021–2024,” version 1.

Because this was a retrospective study based exclusively on clinical record review, no intervention was performed and there was no direct contact with patients. The study involved minimal risk. Confidentiality was ensured throughout the research process by assigning coded identifiers to each record and excluding directly identifying personal information from the analytical database. Access to the database was restricted to the research team, and results were reported in aggregate form to prevent individual identification.

## Results

The analytical sample comprised 146 patients selected according to hospital-stay status, including 73 cases with prolonged hospital stay and 73 controls with non-prolonged stay. Because the study used a prespecified 1:1 case-control sampling ratio, combined-sample descriptive measures were not interpreted as estimates of the underlying hospital population. Patient characteristics are therefore presented according to hospital-stay group in Table 1.

**Table 1:** Characteristics of the analytic sample according to prolonged hospital stay.

Variable	Non-prolonged hospital stay (n = 73)	Prolonged hospital stay (n = 73)	*p* value
Hospital stay			
Length of hospital stay, days	2.0 (2.0–3.0)	6.0 (5.0–9.0)	–

Demographic and anthropometric characteristics			
Age, years	28.0 (22.0–38.0)	36.0 (24.0–45.0)	0.046
Male sex, n (%)	27 (37.0)	40 (54.8)	0.031
BMI, kg/m^2^	24.78 ± 3.75	26.68 ± 4.54	0.006
Obesity, n (%)	8 (11.0)	16 (21.9)	0.074

Preoperative clinical characteristics			
Type 2 diabetes mellitus, n (%)	1 (1.4)	3 (4.1)	0.620
Hypertension, n (%)	1 (1.4)	3 (4.1)	0.620
Harmful habits, n (%)	1 (1.4)	1 (1.4)	1.000
Hemoglobin, g/L	140.5 (128.7–152.3)	148.2 (135.5–160.9)	0.003
Leukocyte count, ×10^9^/L	13.5 (9.0–16.5)	13.6 (10.9–18.4)	0.112
C-reactive protein, mg/L, median (IQR)^1^	18.0 (12.0–48.0)	96.0 (48.0–120.0)	–

Surgical and postoperative characteristics			
Complicated appendicitis, n (%)	20 (27.4)	71 (97.3)	<0.001
Operative time, min	55.0 (40.0–65.0)	85.0 (60.0–130.0)	<0.001
Surgical site infection, n (%)	1 (1.4)	28 (38.4)	<0.001
Pneumonia, n (%)	0 (0.0)	1 (1.4)	1.000
Sepsis, n (%)	0 (0.0)	1 (1.4)	1.000

Note. Data are presented as n (%), mean ± standard deviation, or median (interquartile range), as appropriate. BMI, body mass index; CRP, C-reactive protein. Laboratory variables are reported using SI units: hemoglobin in g/L, leukocyte count in ×10^9^/L, and CRP in mg/L. P values were calculated using Pearson's chi-square test or Fisher's exact test for categorical variables, Student's *t-*test for BMI, and the Mann–Whitney *U* test for age, hemoglobin, leukocyte count, and operative time. No inferential p value was calculated for hospital stay because hospital-stay duration defined case-control status.

1 CRP measurements were available for 13 of 73 patients (17.8%) in each hospital-stay group. Overall, 120 of 146 CRP observations (82.2%) were missing. Numerical CRP measurements were unavailable for all male patients, all patients with obesity, and all patients with surgical site infection. Given this highly selective missingness pattern, CRP values are presented descriptively and no inferential between-group test was performed.

Patients with prolonged hospital stay had a median stay of 6.0 days (IQR, 5.0–9.0), compared with 2.0 days (IQR, 2.0–3.0) among controls. The prolonged-stay group was older, had a higher mean BMI, and included a higher proportion of male patients. Hemoglobin levels were also higher in the prolonged-stay group. In contrast, obesity, type 2 diabetes mellitus, hypertension, harmful habits, and leukocyte count did not differ significantly between the groups.

The largest between-group differences were observed in the surgical and postoperative variables. Complicated appendicitis was more frequent among patients with prolonged stay, operative time was longer, and surgical site infection was substantially more common. Pneumonia and sepsis were uncommon and did not differ significantly between the groups (Table 1).

CRP measurements were available for 13 of 73 patients (17.8%) in each hospital-stay group. The median CRP concentration was 18.0 mg/L (IQR, 12.0–48.0) in the non-prolonged-stay group and 96.0 mg/L (IQR, 48.0–120.0) in the prolonged-stay group. However, 120 of 146 CRP observations (82.2%) were missing, and availability was highly selective. Specifically, no numerical CRP measurements were available for any of the 67 male patients, 24 patients with obesity, or 29 patients with surgical site infection. Therefore, CRP values were reported descriptively without inferential between-group testing, no missing values were imputed, and CRP was not included in the multivariable model.

In the crude logistic regression models, male sex, older age, higher hemoglobin level, complicated appendicitis, surgical site infection, and longer operative time were associated with prolonged hospital stay. After multivariable adjustment, complicated appendicitis and surgical site infection remained associated with prolonged hospitalization. Complicated appendicitis was associated with an adjusted OR of 38.62 (95% CI, 7.78–191.82), while surgical site infection was associated with an adjusted OR of 9.58 (95% CI, 1.14–80.55). Sex, age, hemoglobin level, and operative time did not remain statistically significant after adjustment (Table 2 and Fig. 1).

**Table 2:** Crude and adjusted odds ratios for prolonged hospital stay.

Variable	Crude OR (95% CI)	*p* value	Adjusted OR (95% CI)	*p* value
Male sex (reference: female)	2.07 (1.07–4.00)	0.032	0.86 (0.22–3.44)	0.832
Age, per 10-year increase	1.35 (1.07–1.71)	0.013	1.18 (0.83–1.69)	0.353
Hemoglobin, per 10 g/L increase	1.44 (1.14–1.83)	0.003	1.55 (1.00–2.40)	0.052
Complicated appendicitis (reference: non-complicated)	94.08 (21.07–420.12)	<0.001	38.62 (7.78–191.82)	<0.001
Surgical site infection (reference: no)	44.80 (5.89–340.81)	<0.001	9.58 (1.14–80.55)	0.037
Operative time, per 10-min increase	1.47 (1.26–1.72)	<0.001	1.17 (0.97–1.40)	0.097

Note. OR, odds ratio; CI, confidence interval. Crude estimates were obtained from separate bivariate logistic regression models. Adjusted estimates were obtained from the primary multivariable logistic regression model including sex, age, hemoglobin, type of appendicitis, surgical site infection, and operative time. The outcome was coded as 1 for prolonged hospital stay and 0 for non-prolonged hospital stay; therefore, ORs greater than 1 indicate higher odds of prolonged stay. CRP was excluded because of substantial missingness, while pneumonia and sepsis were excluded because of their very low event counts. A ridge-penalized sensitivity analysis using the same six-covariate set as the primary adjusted model, together with analyses across different regularization strengths, is presented in Supplementary Table S1.

The conventional adjusted estimates for complicated appendicitis and surgical site infection were imprecise, as reflected by their wide confidence intervals. This instability was consistent with the sparse distribution of these variables: only two patients with non-complicated appendicitis were present in the prolonged-stay group, and only one patient with surgical site infection was present in the non-prolonged-stay group.

In the ridge-penalized sensitivity model containing the same six covariates as the primary adjusted model, complicated appendicitis remained positively associated with prolonged hospital stay (penalized OR = 28.75; 95% bootstrap CI, 20.21–41.11), as did surgical site infection (penalized OR = 2.23; 95% bootstrap CI, 1.64–3.06) at C = 1.0. The direction of both associations remained positive across the prespecified range of C values, although the magnitude of the estimate for complicated appendicitis varied substantially with the degree of penalization. These findings were therefore interpreted as a sensitivity analysis of directional robustness rather than as confirmation of the conventional adjusted effect sizes (Supplementary Table S1).

## Discussion

In this study, prolonged hospital stay after appendectomy was associated primarily with complicated appendicitis and postoperative surgical site infection. Patients with prolonged stay also had longer operative times and higher values for some clinical and laboratory variables in the bivariate analysis. However, after multivariable adjustment, only complicated appendicitis and surgical site infection remained associated with prolonged hospitalization.

These findings should be interpreted according to the temporal characteristics of the variables. Complicated appendicitis reflects disease severity established at or before the operative episode and therefore precedes subsequent hospital discharge. In contrast, the timing of SSI onset was not available; consequently, the temporal direction of the association between SSI and prolonged hospital stay cannot be established from the present data.

The definition of prolonged hospital stay varies across studies and clinical settings. In the present study, the institution-specific 75th percentile corresponded to 5 days; therefore, a hospital stay of ≥5 days defined the case group. Other studies have used different thresholds. For example, Cervera-Ocaña and Burgos-Chávez [19] defined prolonged hospitalization as more than 3 days after laparoscopic appendectomy, while Shin et al. [10] defined late discharge as a postoperative stay exceeding 2 days.

Because the present study used a 1:1 case-control sample selected according to hospital-stay status, the combined sample cannot be used to estimate the institutional distribution or typical duration of postoperative hospitalization. We therefore restricted our interpretation to comparisons between the prolonged- and non-prolonged-stay groups rather than comparing the overall sampled length-of-stay distribution with estimates from cohort studies.

Complicated appendicitis showed the strongest adjusted association with prolonged hospital stay. Unlike SSI, complicated appendicitis reflects disease severity established at or before the operative episode and therefore precedes the subsequent duration of hospitalization. This finding is consistent with previous studies, although the magnitude of the association in our analysis was larger. Cervera-Ocaña and Burgos-Chávez [19] reported an OR of 15.01 for prolonged hospitalization after laparoscopic appendectomy, while Zhang et al. [20] reported an adjusted OR of 3.54. Alotaibi et al. [8] also found that complicated appendicitis was associated with hospitalization beyond 72 h, and large multicenter cohorts have reported similar adjusted associations [9], [16].

The larger conventional OR observed in our study probably reflects the concentration of complicated appendicitis among patients with prolonged stay. Only two patients in the prolonged-stay group had non-complicated appendicitis, resulting in sparse cells and a wide confidence interval. In the same-covariate ridge sensitivity analysis, complicated appendicitis remained positively associated with prolonged hospital stay across all evaluated regularization strengths. However, the magnitude of the penalized OR varied substantially according to C. This pattern supports the direction of the association but also reinforces the uncertainty surrounding its precise magnitude in the presence of sparse data.

Surgical site infection was more frequently documented among patients with prolonged hospital stay and remained associated with prolonged stay after multivariable adjustment. Previous studies have similarly reported associations between postoperative infectious complications and longer hospitalization [8], [9], [10], [16]. However, the temporal interpretation of this association requires particular caution in the present study.

The data-collection form recorded SSI as a binary variable but did not capture its date of onset. Consequently, we could not determine whether SSI developed before or after the 5-day threshold used to define prolonged hospital stay. Patients with longer hospitalizations also had a longer period during which an SSI could be detected and documented. Therefore, reverse temporality and differential opportunity for SSI ascertainment cannot be excluded, and SSI should be interpreted as a postoperative condition associated with prolonged hospital stay rather than as an established cause of prolonged hospitalization.

Operative time was longer among patients with prolonged hospital stay but did not remain statistically significant after adjustment. This differs from studies in which operative duration remained associated with prolonged hospitalization after multivariable adjustment. Cervera-Ocaña and Burgos-Chávez [19], Zhang et al. [20], and Shin et al. [10] each reported small but statistically significant increases in the odds of prolonged hospitalization with increasing operative time.

In our study, the attenuation of the association after adjustment suggests that operative time may have served mainly as a marker of case complexity. Longer procedures may reflect complicated appendicitis, adhesions, contamination, technical difficulty, or an increased risk of postoperative infection. Once complicated appendicitis and surgical site infection were included in the model, operative time no longer showed a statistically significant adjusted association.

Baseline and laboratory variables showed some bivariate differences but did not remain statistically significant after adjustment. This partially differs from larger studies in which age, leukocyte count, CRP, peritonitis, appendicolith, and lower hemoglobin levels were associated with prolonged hospitalization [9], [19], [20]. Other studies, however, have also found that age or CRP did not remain significant after adjustment, particularly among patients with uncomplicated appendicitis [10].

CRP could not be evaluated reliably as an associated factor because numerical measurements were available for only 26 patients. Descriptively, CRP values were higher among the available measurements in the prolonged-stay group; however, 82.2% of observations were missing and availability was highly selective. In particular, no numerical CRP measurements were available for male patients, patients with obesity, or patients with surgical site infection. Consequently, no inferential comparison was performed, and CRP was excluded from the multivariable model.

Comorbidities and systemic complications were uncommon and were not statistically associated with prolonged hospital stay. This finding should not be interpreted as evidence that these conditions lack clinical importance. Larger studies have reported associations between prolonged hospitalization and comorbidities, postoperative morbidity, conversion to open surgery, reintervention, and systemic vulnerability [9], [16], [20].

In our sample, only four patients had diabetes, four had hypertension, one had pneumonia, and one had sepsis. These event counts were insufficient to support stable effect estimates. The absence of statistical associations for these variables therefore most likely reflects limited statistical power rather than the absence of clinically relevant effects.

This study provides locally relevant evidence on factors associated with prolonged hospital stay after appendectomy in an Andean public hospital, a setting that remains underrepresented in the international literature. Its main contribution is the identification of two clinically and operationally relevant factors complicated appendicitis and surgical site infection that remained associated with prolonged hospital stay after multivariable adjustment.

From a health-system perspective, these findings support efforts to strengthen early diagnosis, timely surgical management, perioperative infection prevention, and standardized postoperative discharge pathways. Because the study was conducted in a single hospital, the results should not be assumed to apply directly to all settings. Nevertheless, they may be informative for hospitals with similar patient populations, referral patterns, and resource constraints.

This study has several strengths. First, it evaluates a relevant surgical and health-service outcome using real-world data from a public hospital in the Peruvian Andes, a setting underrepresented in the appendectomy literature. Second, the case-control design allowed a focused comparison between patients with prolonged and non-prolonged hospital stay. Third, data extraction was standardized using a structured form, and the pilot review showed high inter-rater agreement.

Several limitations should also be acknowledged. The retrospective design restricted the analysis to information documented in clinical records and may have introduced misclassification or information bias. CRP measurements were available for only 17.8% of the sample. Although availability was identical in the two hospital-stay groups, it differed across other patient characteristics, suggesting that the missingness may not have occurred completely at random. Consequently, CRP was summarized descriptively without inferential testing, no imputation was performed, and the variable was excluded from the adjusted model.

An additional limitation concerns the temporal assessment of surgical site infection. SSI was recorded as a dichotomous variable, and the date of onset was not available in the analytical dataset. We therefore could not establish whether SSI preceded the 5-day threshold for prolonged hospital stay or developed later during hospitalization. Moreover, patients with longer stays had a greater opportunity for in-hospital detection of SSI. Reverse temporality and differential ascertainment are therefore possible, and the observed association between SSI and prolonged hospital stay should not be interpreted as causal. Post-discharge SSI surveillance was not systematically available, which may also have resulted in underascertainment of infections developing after discharge.

Pneumonia and sepsis were too uncommon to support stable effect estimates. In addition, the conventional adjusted ORs for complicated appendicitis and surgical site infection had wide confidence intervals because of sparse cells. Although the same-covariate ridge-penalized sensitivity analysis preserved the positive direction of the associations for complicated appendicitis and surgical site infection across the evaluated regularization strengths, the magnitude of the penalized estimates, particularly for complicated appendicitis, was sensitive to the choice of C. Therefore, the exact magnitude of these associations remains uncertain. Finally, the study was conducted in a single hospital, and its findings should be extrapolated cautiously to other institutions or populations.

## Conclusion

In this retrospective case-control study, complicated appendicitis and surgical site infection remained associated with prolonged hospital stay after multivariable adjustment. The association with complicated appendicitis was consistent with its role as a marker of disease severity present at the operative episode. In contrast, the association with surgical site infection should be interpreted cautiously because the timing of SSI onset was not available, and reverse temporality and differential opportunity for detection cannot be excluded. Older age, male sex, higher BMI, higher hemoglobin levels, and longer operative time showed bivariate differences but did not remain statistically significant in the adjusted model. Strategies aimed at reducing prolonged hospitalization should prioritize timely recognition and management of complicated appendicitis and adherence to perioperative infection-prevention practices, while prospective studies with time-resolved postoperative complication data are needed to clarify the temporal relationship between SSI and length of stay.

## List of abbreviations

BMI: Body mass index
CI: Confidence interval
CRP: C-reactive protein
IQR: Interquartile range
OR: Odds ratio
SD: Standard deviation
SPSS: Statistical Package for the Social Sciences
SSI: Surgical site infection

## CRediT authorship contribution statement

**Irene A. Arias-Romaní:** Writing – review & editing, Writing – original draft, Methodology, Investigation, Data curation, Conceptualization. **Miguel A. Arce-Huamani:** Writing – review & editing, Writing – original draft, Validation, Supervision, Methodology, Investigation, Formal analysis, Data curation, Conceptualization.

## Patient and public involvement

Patients and/or the public were not involved in the design, conduct, reporting, or dissemination plans of this study. The study was based exclusively on retrospective review of clinical records, without direct patient contact.

## Ethics approval

This study was conducted in accordance with the ethical principles of the Declaration of Helsinki and applicable Peruvian regulations for health research. The study protocol was reviewed and approved by the Institutional Committee of Ethics and Scientific Integrity of Universidad Privada Norbert Wiener, Lima, Peru, under approval file No. 0714–2026. Because this was a retrospective study based exclusively on clinical record review, no intervention was performed and there was no direct contact with patients. Therefore, direct informed consent procedures were not applicable. Confidentiality was protected by assigning coded identifiers to the records, removing directly identifying information from the analytical database, and reporting the findings only in aggregate form.

## Declaration of Generative AI and AI-assisted technologies in the writing process

No generative artificial intelligence tools were used to design the study, collect data, perform statistical analyses, or interpret the results. If language-assistance tools were used after manuscript drafting, their use was limited to grammar, clarity, and style editing, and did not generate scientific content. The authors take full responsibility for the accuracy, integrity, and final content of the manuscript.

## Funding sources

This research received no specific grant from any funding agency in the public, commercial, or not-for-profit sectors.

## Declaration of competing interest

The authors declare that they have no conflicts of interest.

## References

[1] Leandri M., Vallicelli C., Santandrea G., Perrina D., Bravi F., Sartelli M. (2025). Postoperative infections after appendectomy for acute appendicitis: the surgeon’s checklist. Antibiotics.

[2] Di Saverio S., Podda M., De Simone B., Ceresoli M., Augustin G., Gori A. (2020). Diagnosis and treatment of acute appendicitis: 2020 update of the WSES Jerusalem guidelines. World J Emerg Surg.

[3] Nguyen A., Lotfollahzadeh S. (2026). StatPearls.

[4] Podda M., Ceresoli M., De Simone B., Fugazzola P., Pata F., Balla A. (2026). Diagnosis and treatment of acute appendicitis: 2025 edition of the World Society of Emergency Surgery Jerusalem guidelines. JAMA Surg.

[5] Ossai C., Wickramasinghe N. (2022).

[6] Li H., Luo T.-F., Zhang N.-R., Zhang L.-Z., Huang X., Jin S.-Q. (2022). Factors associated with prolonged postoperative length of hospital stay after laparoscopic colorectal cancer resection: a secondary analysis of a randomized controlled trial. BMC Surg.

[7] Mărgăritescu N.-D., Mogoantă S.-S., Țenea Cojan T.S., Barbu L.A., Vasile L. (2026). Impact of disease severity and age on outcomes after laparoscopic versus open appendectomy: a single-center retrospective cohort study. Life (Basel, Switzerland).

[8] Alotaibi A.M., Alfawaz M., Felemban L., Moshref L., Moshref R. (2022). Complicated appendicitis increases the hospital length of stay. Surg Open Sci.

[9] Bancke Laverde B.L., Maak M., Langheinrich M., Kersting S., Denz A., Krautz C. (2023). Risk factors for postoperative morbidity, prolonged length of stay and hospital readmission after appendectomy for acute appendicitis. Eur J Trauma Emerg Surg.

[10] Shin J., Ihn M.H., Kim K.S., Kim S.H., Lee J., Yun S. (2023). Risk factors for prolonged hospitalization and delayed treatment completion after laparoscopic appendectomy in patients with uncomplicated acute appendicitis. Ann Coloproctol.

[11] van den Boom A.L., de Wijkerslooth E.M.L., Giesen L.J.X., van Rossem C.C., Toorenvliet B.R., Wijnhoven B.P.L. (2023). Postoperative antibiotics and time to reach discharge criteria after appendectomy for complex appendicitis. Dig Surg.

[12] Martínez-Pérez A., Payá-Llorente C., Santarrufina-Martínez S., Sebastián-Tomás J.C., Martínez-López E., de’Angelis N. (2021). Predictors for prolonged length of stay after laparoscopic appendectomy for complicated acute appendicitis in adults. Surg Endosc.

[13] Alyhari Q., Marzah S., Alsharai H., Alokab S., Al-Wageeh S. (2025). A retrospective cohort study on postoperative surgical site infections following open appendectomy: predictive factors and development and validation of a risk prediction model. Cureus.

[14] Baca D.Z., Vásquez S.R., Reyes E.L. (2024). Características clínico-quirúrgicas de los pacientes peruanos sometidos a apendicectomía. Una revisión sistemática. Rev Cuerpo Med Hosp Nac Almanzor Aguinaga Asenjo.

[15] Mansilla-Sandoval A., Corrales-Delgado D., Puyén Z.M., Mansilla-Doria P., Orendo-Velásquez E., Huicho L. (2025). SARS-CoV-2 infection and complicated appendicitis in adults in Lima, Peru: a matched case-control study. BMC Surg.

[16] Walędziak M., Lasek A., Wysocki M., Su M., Bobowicz M., Myśliwiec P. (2019). Risk factors for serious morbidity, prolonged length of stay and hospital readmission after laparoscopic appendectomy - results from Pol-LA (Polish laparoscopic appendectomy) multicenter large cohort study. Sci Rep.

[17] Arredondo Montero J. (2026). A structured guide to univariate test selection based on normality, variance homogeneity, and graphical data exploration. J Surg Res.

[18] Pepe M.S., Janes H., Longton G., Leisenring W., Newcomb P. (2004). Limitations of the odds ratio in gauging the performance of a diagnostic, prognostic, or screening marker. Am J Epidemiol.

[19] Cervera-Ocaña R.I., Burgos-Chávez O.A. (2023). Factores asociados a la duración de la estancia hospitalaria posterior a la apendicectomía laparoscópica. Rev Colomb Cir.

[20] Zhang P., Zhang Q., Zhao H., Li Y. (2020). Factors affecting the length of hospital stay after laparoscopic appendectomy: a single center study. PloS One.

