# Supplementary material for: Complicated appendicitis and surgical site infection associated with prolonged hospital stay after appendectomy in a Peruvian hospital
Source: Surg Open Sci. 2026 Sep 2;33:100–7. doi: 10.1016/j.sopen.2026.08.004 (PMC13571549; doi:10.1016/j.sopen.2026.08.004)
Supplement: Supplementary file 1 [file mmc1.docx]

**Supplementary Table S1.** Ridge-penalized logistic regression sensitivity analyses for prolonged hospital stay

**Panel A. Same-covariate ridge model at C = 1.0**

| Variable | Penalized OR | 95% bootstrap CI |
| --- | --- | --- |
| Male sex (reference: female) | 0.59 | 0.38–0.90 |
| Age, per 10-year increase | 0.34 | 0.28–0.41 |
| Hemoglobin, per 10 g/L increase | 0.54 | 0.40–0.73 |
| Complicated appendicitis (reference: non-complicated) | 28.75 | 20.21–41.11 |
| Surgical site infection (reference: no) | 2.23 | 1.64–3.06 |
| Operative time, per 10-minute increase | 2.04 | 1.85–2.34 |

**Note.** The ridge-penalized model included the same six covariates as the primary conventional multivariable logistic regression model. Continuous covariates were standardized before penalization and estimates were subsequently back-transformed to their original reporting scales; binary variables retained their 0/1 coding. An L2 penalty with C = 1.0 was used, and the intercept was not penalized. Confidence intervals are percentile-based 95% intervals obtained from 2,000 stratified nonparametric bootstrap resamples, with cases and controls resampled separately within each replicate.

**Panel B. Sensitivity of the main penalized associations to the regularization parameter**

| C | Complicated appendicitis, penalized OR | Surgical site infection, penalized OR |
| --- | --- | --- |
| 0.25 | 7.30 | 1.78 |
| 0.50 | 14.31 | 2.02 |
| 1.00 | 28.75 | 2.23 |
| 2.00 | 57.32 | 2.37 |
| 4.00 | 110.73 | 2.39 |

**Note.** C is the inverse of the regularization strength; therefore, smaller C values correspond to stronger penalization. All models contained sex, age, hemoglobin level, type of appendicitis, surgical site infection, and operative time. The direction of the associations for complicated appendicitis and surgical site infection remained positive across the evaluated C values, although the magnitude of the estimate for complicated appendicitis was sensitive to the degree of penalization.

**Supplementary Table S2. Distributional assessment and selected between-group tests**

| Variable | Shapiro–Wilk *p*, non-prolonged stay | Shapiro–Wilk *p*, prolonged stay | Levene *p* | Selected test |
| --- | --- | --- | --- | --- |
| Length of hospital stay, days | <0.001 | <0.001 | <0.001 | Not tested; defines case-control status |
| Age, years | <0.001 | <0.001 | 0.385 | Mann–Whitney *U* test |
| BMI, kg/m² | 0.111 | 0.037 | 0.125 | Student’s *t* test |
| Hemoglobin, g/L | 0.011 | 0.011 | 0.431 | Mann–Whitney *U* test |
| Leukocyte count, ×10⁹/L | 0.003 | <0.001 | 0.268 | Mann–Whitney *U* test |
| C-reactive protein, mg/L¹ | 0.019 | 0.101 | 0.005 | Descriptive only; no inferential test |
| Operative time, min | 0.013 | <0.001 | <0.001 | Mann–Whitney *U* test |

**Note.** Test selection was based on the combined evaluation of histograms, normal Q–Q plots, Shapiro–Wilk tests, and variance homogeneity rather than on normality-test p values alone. BMI was analyzed using Student’s t test because graphical inspection supported approximate normality, the groups were balanced, and Levene’s test supported equal variances. ¹C-reactive protein was available for only 13 patients in each hospital-stay group. Because 82.2% of observations were missing and availability was highly selective, CRP was summarized descriptively and no inferential between-group test was performed.
